# Non-Tumor Cells within the Tumor Microenvironment—The “Eminence Grise” of the Glioblastoma Pathogenesis and Potential Targets for Therapy

**DOI:** 10.3390/cells13100808

**Published:** 2024-05-09

**Authors:** Aleksandra S. Bugakova, Daria A. Chudakova, Maria S. Myzina, Elvira P. Yanysheva, Iuliia V. Ozerskaya, Alesya V. Soboleva, Vladimir P. Baklaushev, Gaukhar M. Yusubalieva

**Affiliations:** 1 Federal Center for Brain and Neurotechnologies, Federal Medical and Biological Agency of Russia, 117513 Moscow, Russia; 2 Federal Research and Clinical Center of Specialized Medical Care and Medical Technologies Federal Medical and Biological Agency of Russia, 115682 Moscow, Russia; 3 Pulmonology Research Institute, Federal Medical and Biological Agency of Russia, 115682 Moscow, Russia; 4 Engelhardt Institute of Molecular Biology, Russian Academy of Sciences, 119991 Moscow, Russia; 5 Department of Medical Nanobiotechnology of Medical and Biological Faculty, Pirogov Russian National Research Medical University, Ministry of Health of the Russian Federation, 117997 Moscow, Russia

**Keywords:** cancer, glioblastoma, GBM, cancer stem cells, CSCs, tumor microenvironment, novel therapeutic targets, TILs, CAFs, TANs

## Abstract

Glioblastoma (GBM) is the most common malignancy of the central nervous system in adults. GBM has high levels of therapy failure and its prognosis is usually dismal. The phenotypic heterogeneity of the tumor cells, dynamic complexity of non-tumor cell populations within the GBM tumor microenvironment (TME), and their bi-directional cross-talk contribute to the challenges of current therapeutic approaches. Herein, we discuss the etiology of GBM, and describe several major types of non-tumor cells within its TME, their impact on GBM pathogenesis, and molecular mechanisms of such an impact. We also discuss their value as potential therapeutic targets or prognostic biomarkers, with reference to the most recent works on this subject. We conclude that unless all “key player” populations of non-tumor cells within the TME are considered, no breakthrough in developing treatment for GBM can be achieved.

## 1. Introduction

The most prevalent malignancy of the central nervous system (CNS) in adults is glioblastoma, also known as glioblastoma multiforme (GBM), or high-grade glioma, or grade IV astrocytoma [1]. The vast majority of GBM cases (~90%) are the IDH wild type (IDHwt), i.e., they do not harbor pathogenic mutation in the gene encoding isocitrate dehydrogenase (IDH), while around 10% of cases is IDH-mutated GBM, which is known to have a better prognosis for patients [2]. GBM is highly aggressive and currently incurable, with the average median survival rate less than 15 months after the standard of care treatment [3], and the 5-year survival rate is less than 5% [4]. Such a dramatic outcome can be explained by extreme drug resistance and a high rate of tumor recurrence (~80% of cases) of GBM [5,6,7]. Despite numerous studies, it is still not completely clear how GBM occurs.

The dominant theory of the origin of the GBM tumor is a cancer stem cell (CSC) theory. It postulates that in the case of the GBM, the tumor originates from CSCs derived from neural stem cells (NSCs) or neural progenitor cells (NPCs) located in the neural stem cell niche in the subventricular zone (SVZ) [8]. CSCs are capable of self-renewal, multilineage differentiation, and tumor initiation. There are two major models within the CSC theory of GBM, “vertical/hierarchical” and “horizontal”. The “hierarchical model” postulates that CSCs are immortalized cells at the apex of the very rigid hierarchy of the tumor, capable of self-renewing and also giving rise to progeny of all tumor cell lineages [9]. Despite comprising only a small fraction of the whole tumor cell population, they are considered key drivers of GBM. A “horizontal” theory is based on the data showing that inside the tumor mass, some cells may dedifferentiate [10], obtain a stem-like phenotype, and subsequently give rise to a heterogeneous cancer cell progeny.

The major challenges for GBM treatment are (1) tumor localization and consequently challenges for drug delivery; (2) an immunosuppressive tumor microenvironment (TME) with low cytotoxic lymphocyte infiltration; (3) high intra- and intertumor phenotype heterogeneity; (4) a low mutational burden and hence a lack of neoantigen and tumor-specific antigen presentation; (5) the existence of the therapy-resistant glioblastoma stem-like cells; and (6) dynamic tumor plasticity.

A recent single-cell RNA-sequencing (scRNAseq) study revealed four major phenotypes of GBM cells, namely oligodendrocyte progenitor-like (OPC-like), neural progenitor-like (NPC-like), astrocyte-like (AC-like), and mesenchymal-like (MES-like) [11]. Such states are suggested to be dynamic and influenced by the tumor microenvironment (TME). Notably, a “mesenchymal-like” transcriptional signature is a characteristic of GBM cells that facilitates immune evasion and is supposedly acquired by cells after an immune attack [12]. Furthermore, the fetal brain “roadmap” constructed based on the scRNAseq dataset and projected onto the scRNAseq data obtained from patient-derived GBM samples revealed that GBM at least partially mimics brain development and includes cells with the features of the three common cell lineages specific to the developing brain, namely astrocytic, neuronal, and oligodendrocytic [13]. However, despite significant and fast advances in our understanding of the GBM origin, it is still far from being complete. Thus, further investigations on this topic are needed to determine molecular mechanisms allowing the TME to modulate such phenotypic switches of the GBM cells. This will undoubtedly lead to the identification of the novel therapeutic targets, thus increasing efficiency of anti-CSC therapy in GBM.

Currently, the mainstay of GBM therapy is the, formulated in 2005, standard of care based on the maximal safe surgical resection of the tumor, followed by chemotherapy and radiotherapy [3]. New approaches to radiotherapy and electric field therapy, such as proton beam therapy or tumor-treating fields, have been proposed for GBM treatment [14,15]. As for the chemotherapy, several alkylating agents such as temozolomide (TMZ) [10], carmustine (BiCNU) [16], and lomustine (CCNU) [17] have been reported to lead to prolonged overall survival (OS) of patients with GBM. Furthermore, certain epigenetic features may determine the outcome of GBM chemotherapy, advocating for the personalized therapy approach based on DNA methylation tests. For instance, the methylation of the promoter region of the gene MGMT encoding O-6-methylguanine-DNA methyltransferase results in compromised DNA repair and is not only an independent good prognosis factor in GBM, but is also associated with a better response to TMZ treatment in combination with radiotherapy compared to radiotherapy only [18].

Although the standard of care has not changed significantly for almost two decades, several additional therapeutic strategies for GBM have been proposed, such as various oncolytic virus therapies; therapies by monoclonal antibodies (MABs) including those targeting angiogenesis; therapies targeting the tumor extracellular matrix (ECM) and ribosome biogenesis; approaches based on metabolic dependences of the GBM; epigenetic-based therapies; etc. [19,20,21,22,23,24]. The cancer stem cells (CSCs) within the GBM are particularly attractive targets for therapy [25], given their potential leading role in tumor chemoresistance, disease recurrence, and cancer cell phenotype plasticity, as demonstrated in GBM and other types of cancer. Finally, the tumor microenvironment (TME) is also rapidly gaining interest of translational oncologists as a potential therapeutic target or a source of diagnostic and prognostic markers, especially given its “cross-talk” with CSCs. Such cross-talk is bi-directional, with CSCs affecting immune cells and the immune response and vice versa, at least in other types of cancer, making CSCs an attractive target for immunomodulation [26,27].

**Figure 1:**
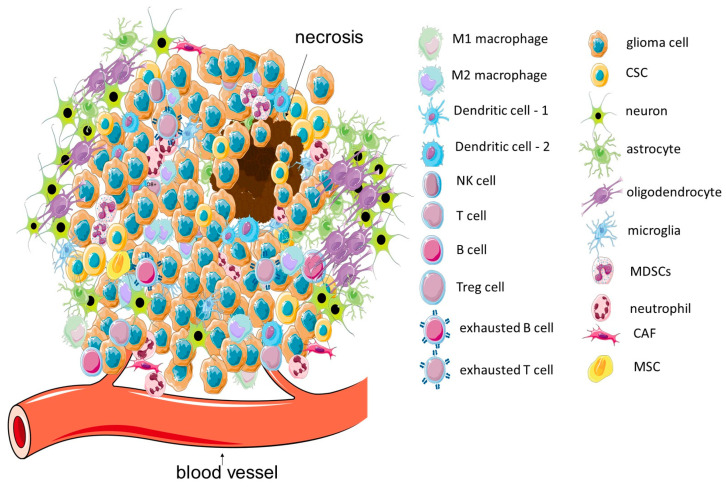
Different non-tumor cell populations within the GBM TME. Some elements of the figure were created using Server Medical Art figures licensed under a Creative Common Attribution 3.0 Unported License; https://smart.servier.com (accessed on 2 March 2024). Some elements were created with the use of free figures from https://bioicons.com (accessed on 2 March 2024) under a Creative Common license.

The GBM TME is ever-changing depending on the stage of disease or in response to the therapy. It has a high degree of complexity and comprises several types of cancerous and non-cancerous cells (astrocytes, pericytes, neuronal progenitor cells, endothelial cells, and other residential brain cells, as well as a variety of immune cells—either resident or recruited—such as microglia, dendritic cells, and leukocytes) (Figure 1). It also includes components of the ECM and dynamically changing cellular secretome (extracellular vesicles (EVs) and their molecular cargo, ECM-modifying enzymes, cytokines, metabolites, etc.) [24,28,29]. Despite the fact that GBM is considered a “cold” neoplasm (tumor with high levels of pro-tumor and low levels of anti-tumor immune cells), the modulation of immune cells’ behavior within the GBM TME is a promising therapeutic tool.

To name but a few, chimeric antigen receptor (CAR)-T cell therapy [30], tumor neoantigen vaccines in combination with immunomodulators [31], and immunotherapies targeting CSCs in GBM [32] have been developed recently. Having said this, we also point to the challenges in GBM immunotherapy; an example is limited efficacy of a PD-1/PD-L1 checkpoint blockade [33], although targeting the PD-1/PD-L1 axis may still confer certain benefits [34].

Undoubtedly, deciphering the molecular mechanisms that regulate the immune response within the GBM TME will allow the development of novel, more efficient therapeutic treatments.

There are many comprehensive reviews on this subject published so far [35,36,37,38], but the number of works in the field grows rapidly, warranting the need for an up-to-date review incorporating the newest developments and trends. In our concise narrative review, we characterize several populations of cells within the GBM TME with particular focus on immune cells. We omit describing in detail the role of the cells composing the tumor vasculature, given the wealth of literature on this subject [39], but make a note here that in relation to the migration of immune cells to the GBM tumor site and their redistribution and activity within the TME, vascular cells obviously play a role.

## 2. GBM Microenvironment: Tumor Localization and Immune Features of TME

For several decades, the brain was considered an immunoprivileged zone, suggesting that xenotransplants would not be rejected by the immune system, and brain tumors will evade immune surveillance. However, more recent studies have demonstrated that immune cells can be found in brain parenchyma, such as regulatory T cells (Tregs), resident macrophages, and microglia, and they maintain an immunosuppressive microenvironment [40]. In “healthy conditions”, the BBB restricts the entry of immunocompetent cells into the brain from the bloodstream [40]; in the case of inflammation or malignancy, or following therapeutic interventions such as radiotherapy, the BBB becomes compromised, and lymphoid and myeloid cells can enter the brain tissue. For example, during ischemia, which may occur in the necrotic core of GBM, microglia within the TME start to produce proinflammatory cytokines such as interleukin 1 beta (IL-1β) and tumor necrosis factor alpha (TNFα) [41], thereby initiating BBB damage. The dysfunction of BBB is one of the hallmarks of GBM. Speaking of GBM therapy, increased permeability of BBB will facilitate both drug delivery to the tumor and the infiltration of the TME with effector immune cells. However, that usually occurs in the late stages of the disease when the therapy is not effective and the tumor is highly aggressive.

## 3. Neural Cells in GBM TME

### 3.1. Astrocytes

Astrocytes are the glial cells that constitute around 20–40% of total cell mass in the adult human brain [42]. In physiological conditions, they regulate fluidics flow via participating in the formation of the blood–brain barrier (BBB) [43], maintain synaptic transmission via removing neurotransmitters from the synaptic cleft [44], and supply neurons with glucose under hypoglycemia conditions [45]. This is just a short summary of the major physiological functions of astrocytes in the CNS; for more detailed review, we refer the readers to the recent work of Zhang et al. [46].

In the case of brain lesions (including tumor), astrocytes go into a reactive state and “isolate” damaged tissue from the healthy ones. Reactive astrocytes can be characterized as hypertrophic, highly proliferative cells with an increased expression of intermediate filaments such as glial fibrillary acidic protein (GFAP), vimentin, and nestin; transcription factors (STAT3); growth factors (GDF-15 and BDNF); inflammatory cytokines (IL6 and CCL2); extracellular matrix proteins (collagen); and cell adhesion protein (CD44), indicating their role in the reparative process [47]. In recent work, Watson et al. [48] investigated organelle transfer in GBM tissue. They revealed that GBM cells can receive mitochondria from adjusted astrocytes through tumor microtubes. Tumor cells with transferred astrocytic mitochondria showed higher proliferation capacity (bigger proportion of tumor cells in G2/M phase of cell cycle) and capability to self-renew (higher expression of SOX2 and OCT4) compared with GBM cells without mitochondria transfer. Importantly, they additionally determined that the inhibition of growth-associated protein 43 (GAP43), which is responsible for the formation of tumor microtubes in GBM cells and in astrocytes, leads to decreased mitochondria transfer. Therefore, GAP43 can be considered as another potential therapeutic target for GBM treatment. Interestingly, the organelle transfer seems to be bidirectional. In another recent study, the role of tunneling nanotubes (TNTs) in GBM was investigated as well [49]. The authors demonstrated that TNTs serve as a “highway” for GBM mitochondria transfer to astrocytes. The enlarged tumor mitochondria were detected in tumor-associated astrocytes via time-lapse microscopy, and GBM mDNA was isolated from such astrocytes. Additionally, it has been shown that the transfer of GBM mitochondria converted TME astrocytes’ metabolism into a more tumor-like metabolism; moreover, astrocytes harboring tumor mitochondria seemed to be more resistant to hypoxic conditions. Thus, TNTs might be a novel therapeutic target in GBM, given that TNTs are found not only in vitro but also in vivo as well as in primary GBM tumors. Moreover, it has been shown that radiation and chemotherapy promote TNT formation in GBM and are associated with the transfer of antiapoptotic protein MGMT from resistant to sensitive tumor cells. This leads to development of resistance of GBM cells to radio- and/or chemotherapy [50].

Another work focusing on interaction between astrocytes and microglia demonstrated that tumor-associated astrocytes (TAAs) express high levels of CD274 (PDL-1), CHI3L1, and STAT3, which are associated with alternatively activated astrocytes [51].

The role of astrocytes in the GBM TME is very diverse. For instance, in a study of Mega et al. [52], the impact of astrocytes on GBM cell proliferation was assessed. It was shown from the analysis of open-access database TCGA GBM that a high “astrocytic signature” is associated with lower survival in patients. The co-cultivation of a GBM cell line (U251) with human astrocytes resulted in an elevated proliferation of tumor cells. From a point of view regarding an immune TME, it has been shown that astrocytes affect the immune niche of the GBM via the CCL2- and CSF1-governed recruitment of tumor-associated macrophages (TAMs) and promotion of their pro-tumorigenic phenotype [53]. It is not the only example of the immunomodulatory role of astrocytes in brain tumors [54] and in GBM in particular [55]. Additionally, molecular cargo of extracellular vesicles (EVs) secreted by GBM cells may promote the neoplastic transformation of premalignant astrocytes [56]. Finally, it has been found recently that irradiation-induced senescent astrocytes play a substantial tumor-promoting role after radiotherapy. In preclinical models of the aforementioned scenario, senolytic agents substantially prolonged the time of survival [57].

### 3.2. Oligodendrocytes

Oligodendrocytes (OLCs) are the cells supporting the myelin sheath in the CNS. Depending on the differentiation stage, they can be subdivided into oligodendrocyte precursor cells (OPCs), newly formed oligodendrocytes (NFOs), and myelinating oligodendrocytes (MYOs). It appears that OLCs take part in radio- and chemoresistance and consequently recurrence of GBM, as an A172 GBM cell line cultivated in OLC-CM had a higher expression of efflux transporter ABCG2 partly responsible for drug resistance [58,59,60]. In the context of the immune component of the TME, it was reported recently that inflammatory cytokines can “convert” oligodendroglia to the antigen-presenting-cell-like state in multiple sclerosis [61]. Given that chronic inflammation is a characteristic feature of the GBM TME, it is plausible to suggest that such conversion may take place in GBM too. Most likely, other types of cell–cell interactions between GBM cells, neural cells, and immune cells within the TME, yet to be characterized, also contribute to GBM development. As for CSCs, it was shown that GBM cells cultured in vitro in an OLC-conditioned medium (OLC-CM) had a higher expression of stemness factors such as SOX2, Nanog, Oct3/4, ALDH1, and Bmi1 compared with the control group [59]. Finally, it was demonstrated recently that a microenvironment composed of brain white matter and “injured” by the tumor invasion triggers the differentiation of GBM cells (those with oligodendrocyte competency) towards the oligodendroglial lineage, in a SOX10-dependent manner. Thus, myelination-promoting compounds and compounds that increase levels of SOX10 might be a potential therapeutic tool for a subset of GBM with pro-oligodendrocyte differentiation potential [62]. To summarize, it appears that cells of an oligodendrocyte lineage can either promote GBM CSCs’ stemness or shift them towards differentiation, depending on other factors yet to be characterized.

Here, we also note that with regard to neural cells in the GBM TME, an interaction of non-malignant neurons with GBM exists and plays a significant role in GBM pathogenesis, described in detail in a recent comprehensive review by Sharma et al. [28].

## 4. Innate Immunity Cells in GBM TME

Myeloid cells, namely tumor-associated macrophages (TAMs), myeloid-derived suppressor cells (MDSCs), dendritic cells (DCs), and tumor-associated neutrophils (TANs), are the major contributors both to immunosuppression and tumor progression in GBM; thus, they are expected to have a therapeutic potential in GBM [63]. Here, we describe their role in a concise manner, with the focus on their potential translational value.

### 4.1. Tumor-Associated Macrophages: Microglia and Bone-Marrow-Derived Macrophages

Tumor-associated macrophages (TAMs)—resident macrophages (microglia) and bone-marrow-derived macrophages (BMDMs)—are the predominant non-cancer cell populations within GBM, comprising around 30–40% of the total cell mass. These two populations are distinct cell types; microglia originate from early hematopoietic progenitor cells and migrate from the yolk sac, whereas BMDMs originate later during development from the bone marrow [64]. Microglia are cells expressing CD11b+/CD45low (or CD45int), and bone-marrow-derived macrophages (BMDMs) are the population of cells with phenotype CD11b+/CD45 high [65]. Macrophages may possess pro-tumor as well as anti-tumor activities (so called “cold” M2 and “hot” M1 phenotypes, respectively), although such a view is too simplistic, and recent data suggest that their “true” phenotype in vivo is closer to a dynamic spectrum among M0, M1, and M2 phenotypes with numerous intermediate stages [55]. It has been shown that GBM-infiltrated innate immune cells resemble M0 macrophages [66]. As for their localization, it appears that M1 cells are predominantly found in the necrotic core of the tumor, whereas M0–M2 cells are found in the periphery of the tumor [67]. According to a classical view, in the case of inflammation, macrophages and microglia will work as antigen-presenting cells and phagocytes. Additionally, they maintain anti-tumor immunity through antibody-dependent cell phagocytosis (ADCP) in the presence of tumor-specific antibodies and eliminate cells expressing so-called “eat-me” molecules that are markers of apoptosis [68]. However, tumors usually produce a broad range of anti-inflammatory molecules, such as TGF-ß, that repolarize macrophages and microglia into a more “regenerative/wound healing” state (M2-like). That leads to the secretion of anti-inflammatory cytokines (IL6, IL10, and TGF-ß) [69] and inhibition of effector T cells, remodeling of the ECM via matrix metalloproteinases and promotion of tumor cell mobility [70], stimulation of neoangiogenesis through VEGF and CXCL2 pathways [71], and stimulation of tumor proliferation through the EGF-EGFR axis [72]. The role of the TAM in GBM is reviewed in numerous works elsewhere [73,74], and several clinical studies are being conducted right now, aiming to increase their anti-tumor activity [64] or to decrease M2 infiltration into the GBM tumor site [75]. In addition, macrophages are also used as CAR carriers and currently are tested in clinical trials against several solid tumors [NCT04660929, NCT05007379]. It should be noted that GBM-driven macrophage activation is supposedly patient-specific; for example, the secretome from two different GBM cell lines triggered distinct transcriptional responses of macrophages in vitro, suggesting patient-specific patterns of TAM activation [76]. This advocates for more personalized approaches to immune modulation in GBM.

### 4.2. Myeloid-Derived Suppressor Cells

Myeloid-derived suppressor cells (MDSCs) are a heterogeneous group of myeloid progenitors (defined as cells with phenotype CD11b+CD33+HLA-DRlow/- in humans [77] and CD11b+Gr1+ in mice [78] that differentiate into monocytes or granulocytes under normal conditions). They produce immunosuppressive molecules such as transforming growth factor β (TGFβ) and interleukin-10 (IL-10), resulting in the subsequent suppression of T and B effector cells and NK cells and the accumulation of regulatory T cells (Tregs) [79]. MDSCs within the GBM TME can be subclassified into granulocytic or polymorphonuclear MDSCs (G/PMN-MDSCs), monocytic MDSCs (M-MDSCs), and early-stage MDSCs (eMDSCs). Their emerging roles in GBM are summarized elsewhere [79,80]. In a study of Dubinski et al. [81], it was shown that an increased proportion of granulocytic MDSCs in the GBM TME is associated with the suppression of CD4+ T effector cells through the upregulation of the PD1-PDL-1 axis. Another recent study showcased the anti-immunosuppressive effect of IL15-armored CAR-T cells in GBM models [82]. Firstly, the authors examined the expression of IL15Ra in MDSCs and found its abundant expression. The second step was to assess the impact of IL13Ra2 CAR-T cells armored with secreting IL15 (CARIL15s) or directly fused with CAR protein (CARIL15f). It was found that both models promote survival of mice compared with simple CAR-T therapy, while CARIL15f showed a higher anti-tumor effect. The effect of armored CAR-Ts was associated with the depletion of MDSCs or their immunosuppressive dysfunction (decrease in IL10 and TGF-b). The prevalent effect of fused IL15 CAR structures can be explained by direct cytotoxic activity against MDSCs. Moreover, in CAR-IL15-treated mice, promoted CD8+ T cell, NK cell, and B cell infiltration was observed [82]. In another study, it was shown that macrophage migration inhibitory factor (MIF) is mainly produced by cancer stem-like cells and attracts MDSCs. That leads to the upregulation of arginase-1 expression and inhibition of cytotoxic lymphocytes (CD8+) [83].

Interestingly, the predominant monocytic MDSC infiltration was detected in the GBM TME [84]. These studies reveal different pathways that might be considered as potential therapeutic targets.

### 4.3. Tumor-Associated Neutrophils

Neutrophils are the most numerous circulating leukocytes, defined as CD66b+, CD15+, CD14−, and CD33+ cells (in humans). The main functions of neutrophils are the regulation of inflammation and response to microbial threats. Based on their role and phenotype, tumor-associated neutrophils (TANs) can be subdivided into anti-tumor/immunostimulating (N1) and pro-tumor/immunosuppressing (N2) cells [85]. Typically, TANs associated with GBM have a T cell immunosuppressive, pro-angiogenic phenotype [86]. A novel finding is the role of NET in promoting Treg formation in GBM [87]. To avoid redundancy, we refer readers to recent comprehensive reviews on the role of neutrophils in glioma and brain metastasis [88,89]. Here, we highlight the emerging concept of the significance of TANs as biomarkers of GBM. For example, it has been demonstrated that a high tumor infiltration of TANs before radiotherapy is a prognostic marker of poor overall survival in GBM [85]. Finally, an interesting approach is to use neutrophils as a therapeutic tool rather than a target, as was demonstrated in a recent study in which CAR-neutrophils were loaded with nanodrugs for their targeted delivery to GBM cells [90]; other examples of using neutrophils for drug delivery for GBM therapy have been described recently [91].

### 4.4. Dendritic Cells

Mature dendritic cells (DCs) can be divided into three subpopulations: conventional DC type 1 (cDC1), conventional DC type 2 (cDC2), and plasmacytoid DC (pDC). The major difference between types is the type of T cells they prime: cDC1 is known to cross-present antigens on the surface of MHCI, thereby activating CD8+ T cells, while cDC2 is known to present antigens via MHCII and prime T helper cells and Tregs [92]. The role of conventional DCs is to process and present antigens for further priming of T cell and B cell activity [93]. In the context of cancer, pDCs possess proinflammatory activity (IFN I production and antigen presentation) as well as pro-tumorigenic activity (secretion of IL10, TGFb, and checkpoint ligands suppressing CD8+ T cell activity) [94,95]. cDC1 cells besides cross-presentation produce IL12, promoting CD8+ T cell polarization into cytotoxic cells [95,96,97]. While under normal conditions, DCs are present in a small proportion in the brain (choroid plexus and meninges), in inflammatory conditions, the infiltration of DCs increases and is usually associated with a better GBM prognosis [98]. As for an immunotherapeutic approach, the idea to ex vivo load DCs with tumor-associated antigens and reinfuse them back to patients is promising. Finally, to name but a few, currently, a phase III clinical study of a DC vaccine is under investigation for patients with GBM [99,100].

## 5. Semi-Innate Immunity Cells in GBM TME

### 5.1. Natural Killer Cells

Natural killer cells (NKs) have a rather unique role in the anti-tumor immunity because they are not restricted to recognizing tumor-specific antigens (this might be particularly important in the case of highly heterogeneous tumors), but can recognize the lack of the expression of MHC I complexes (for example, KIR-mediated), recognize AB-opsonized cells through FcRγIIIa (antibody-mediated cytotoxicity), and recognize stressed cells via MICA, MICB, and ULBP family proteins binding to NKG2D receptors [101,102]. Their presence in tumor tissue is documented, indicating their ability to penetrate GBM tissue [103]. Thus, NK cells are considered carriers of chimeric antigen receptors for targeted immunotherapy. However, even if the patient does not have a Graft-versus-Host reaction, they still may have a host-vs.-graft reaction, and that can lead to short-term persistence of transfused cells [104]. The anti-GBM role of NK cells is comprehensively discussed in the recent review of Sedgwick et al. [105], and here we would like to mention only some of their key features relevant to GBM CSCs. It was shown that NK cells both possess cytotoxic activity against CSCs and promote their differentiation [106,107]. Since differentiated GBM cells are more susceptible to chemotherapy, the combination of NK cell therapy and chemotherapy might be considered as a potential therapy of GBM.

However, NK cells introduced to GBM cells (and GCSs specifically) seem to adopt an altered phenotype with decreased cytotoxicity. It was established that direct cell–cell contact is an essential part in the suppression of NK cell functioning. One of the possible axes involved in that is the TGFb/av-integrin axis. In a recent study of Shaim [108], it was shown that engagement of CD103 and/or CD9 on the surface of NK cells with the integrin (CD51) on the surface of GSCs promotes TGFb expression by GSCs. TGFb, in turn, changes the phenotype of NK cells (leads to downregulation of activatory receptors and co-receptors such as NKG2D, CD16, and DNAM1 and upregulation of inhibitory receptors such as TIM3). Therefore, the inhibition of the TGFb/av-integrin axis is expected to promote NK cytotoxicity and survival. The immunoinhibitory role of TGBb in GBM was also showcased in another recent study [109]. The ex vivo expansion of auto- or allogeneic NK cells and their reinfusion seems to be beneficial for patients with GBM [110,111]. Additionally, TGFb can be negated by the introduction of dominant negative TGFb receptors in NK cells to be used for therapy [111]. An alternative approach to NK-cell-based therapy is CAR-NK treatment. For instance, antiHER2-CAR NK therapy was investigated in a phase I clinical trial on patients with GBM and revealed higher CD8+ T cell infiltration and the “stabilization of disease” in some of the participants [112]. Recently, we made an attempt to improve NK cell anti-tumor activity by enhancing NK cells via *CISH* gene knockout [113], based on the fact that the protein encoded by *CISH* is known to inhibit NK cell susceptibility to IL15 and, consequently, their cytotoxic activity. A promising combinatorial approach is to promote NK and T cell survival via co-transferring them with an IL15/Il15R-expressing oncolytic virus [114].

### 5.2. Natural Killer/T Cells

Natural killer/T cells (NKT cells) are another small subpopulation of TILs in the GBM TME [115]. There are two distinct types of NKT cells: invariant (express semi-invariant TCRa chain in combination with limited TCRb chain, Vb11 in humans) and variant NKT cells (express variant TCR similar to conventional T cells) [115,116]. Speaking of their anticancer activity, NKTs have both direct and indirect functions: the formation and secretion of perforins and granzymes, via the TCR-ag-CD1d complex, or activation and secretion of anti-tumor cytokines such as INFy (activation of other populations of cytotoxic cells). The recognition of the cognate (lipid) antigen is mediated by the CD1d MHC-like molecule, which was shown to be relatively highly expressed in GBM cells [117]. NKTs are “restricted” to the CD1d molecule and predominantly recognize lipid antigens presented by CD1d. That property may be significant in the case of a brain tumor due to the high proportion of lipid (sphingolipids, cholesterol, etc.)-surface-molecule-derived antigens [118]. It is known that disialoganglioside 2 and 3 (GD2, GD3) are abundant in GBM and neuroblastoma cells and almost not present in healthy cells [119,120]. The recognition of lipid antigens and direct cytotoxicity foreshadow the potential anti-GBM activity of NKT cells [115]. Currently, the clinical study assessing GD2-CAR NKT cells for neuroblastoma therapy is in the stage of patient recruitment [NCT03294954]. Specifically, GBM expressions of lipid antigens such as GD2 and GD3 are increased, determining potential activity of iNKT cells [121]; in a relevant GD1D-expressing GBM xenograft model the direct intracranial injection of human NKT cells showcased efficacy [117].

## 6. Adaptive Immunity Cells in GBM TME—T Cells

### 6.1. CD8+ T Cells

Cytotoxic T lymphocytes (CD8+ T cells) have proven to be a valuable tool for cancer immunotherapy. Through the direct recognition of the tumor-specific or tumor-associated antigens/MHC I complexes on the surface of tumor cells, cytotoxic CD8+ T cells become activated and start to release granzymes and perforins that kill target cells [122]. The presence of CD8+ T cells in tumor parenchyma may indicate anti-tumoral adoptive immunity. However, due to a highly immunosuppressive TME, caused by an immunosuppressive cytokine milieu/expression of the immunosuppressive molecules (for example, IL10, TGFß, PD-1L, TIM3-L, CTLA4-L, and LAC3-L) [81], it is more likely that T effector cells within a GBM tumor lesion are suppressed. The promotion of CD8+ T cell migration towards the TME and modulation of PD-1/PD-L checkpoint ligands via Immune Checkpoint inhibitors (ICIs) are supposed to result in tumor regression. However, recently conducted clinical trials of GBM CAR-T or ICI have not shown prolonged survival in patients with GBM [123]. As we can assume, the presence of cytotoxic cells is not always associated with their activity due to the high immunosuppressive function of GBM cells and its TME. T cell dysfunction or “exhaustion” is usually explained by the expression of inhibitory molecules such as PD1, TIM3, LAG3, and CTLA-4. In the recent scRNA seq study, it was established that T cells, spatially co-localized with HMOX1+ (immunosuppressive phenotype) myeloid cells in mesenchymal-like areas of GBM [124], express markers of dysfunction, and their suppression is associated with IL10 present in the TME. Subsequently, the inhibition of the JAK/STAT pathway (regulated by IL10) resulted in the rescue of T cell function in in vitro and in vivo settings [125]. In line with this, a case study report documented an increase in CD8+ T cells in a GBM specimen post resection after Ruxolitinib (JAK/STAT inhibitor) treatment. Interestingly, the shifting to effector or effector memory states in both CD4+ and CD8+ T cell populations was observed. The patient was alive 24 months after therapy at the time of the publication of the case report [125]. An alternative mechanism by which GBM may escape immune surveillance is the sequestration of naive T cells in the bone marrow managed though the internalization of S1P1 from the surface of T cells limiting their migratory activity to the tumor tissue [126]. Thus, the stabilization of S1P1 on the surface may be a possible therapeutic approach. Interestingly, the composition of immune cell populations within the GBM tumor depends on their localization. In a recent study of Tamura et al. [127], it was shown that a lack of CD8+ T cells and abundance of Tregs and TAMs and PD1+CD8+ T cells was inside the tumor core, probably due to the hypoxic environment. Interestingly, CD8+ T cell “exhaustion” in GBM appears to be sex-biased, and in particular it is more prominent in male patients with GBM [128]. As for the (CAR)-T therapy of GBM (adoptive cell transfer of ex vivo expanded and transformed T cells), several surface markers of GBM are currently being used for development of CAR proteins, namely NKG2D [129], EGFRvIII [130], IL13Rα2 [131], HER2 [132], GD2 [133], etc. Results of corresponding clinical trials are comprehensively reviewed elsewhere [134]. Even though efficacy was shown in GBM preclinical models for the majority of these targeted (CAR)-T trials (NCT02664363, NCT01454596), no significant therapeutic effects have been observed yet.

### 6.2. Gamma Delta (γδ) T Cells

Gamma delta (γδ) T cells are small subpopulations of non-conventional T cells known for their proinflammatory capability executed via antigen recognition in an MHC-independent context and expression of multiple cytokines such as GM-CSF, IL-4, IL-17, IL-21, IL-22, and IFN-γ [135]. It was shown that the (γδ) T cell presence is predominantly associated with a better prognosis in patients with cancer [136]. Interestingly, in one of the recent studies [137], a GBM-specific γδ-T cell clonotype was established in three out of four patients (Vγ9Jγ2-Vδ2), indicating GBM-specific antigen recognition by this small subpopulation of T cells. In the case of cell-based therapy of GBM, (γδ) T cells seem to be a suitable subset of T cells due to their capability to infiltrate tumor tissue and anti-tumor activity (perforin–granzyme-mediated, expression of IFN-γ and TNFα) [138]. However, the GBM TME could be suppressive for these cells as well. It was shown that the anti-GBM activity of (γδ) T cells is diminished in hypoxia settings through the activation of the kinase A pathway, leading to a decreased expression of the activatory NKG2D receptor [121]. The anti-tumor functions of (γδ) T cell subpopulations are wide and under active investigation, and we suggest to refer to the recent reviews on this subject [139,140].

### 6.3. MAIT T Cells

Another small population of (αβ) invariant T cells is MAIT (mucosal-associated invariant T cells) that recognizes derivatives of riboflavin biosynthesis [141]. Their function is mainly associated with antibacterial immunity [142] in mucosal tissue. However, the combination of both innate and adaptive immunity features, long-term persistence, and the absence of Graft-versus-Host reactivity makes them an attractive tool for cell-engineering-based approaches in anti-tumor therapy (CAR-MAIT cells) [143].

### 6.4. CD4+ T Helper Cells

The activation of antigen-specific immunity is regulated by CD4+ T helper (Th) cells that express antigen receptors recognizing the fragments of antigens in complex with MHC-II molecules, although they have a multitude of other functions. The total population of Th cells is subdivided into several main subclasses (Th1, Th2, and Th17); all of them can be found in GBM [144]. Generally, it is assumed that Th1 cells predominantly have higher potency of anti-tumor activity among other T helper phenotypes [145], while the role of Th2 remains ambiguous. It was shown that low Th2 and low activity of the PD-L1/PD-1 is a predictor of a good prognosis [146]. Notably, it was reported recently that GBM stimulates tumor-infiltrating CD4+ T cells to shift towards the Th17 phenotype [147]. Thus, the pathways guiding such a shift might be good therapeutic targets. However, the association between the T helper phenotype and anti-tumor activity remains to be investigated [148]. The intimate interaction among antigen-presenting cells (APCs)/Th cells/T effector cells/B cells is essential for efficient and long-term anti-tumor immunity [144]. In the case of GBM, there is a phenomenon of so-called Th “exhaustion” (progressive loss of function), presumably diminishing the anti-tumor activity of the mixed population of CD4+ T cells. However, there also are data demonstrating such “exhaustion” only for CD8+, rather than for CD4+ T cells [149]. Determining the precise molecular mechanisms of the T cell “exhaustion” [150] in GBM, defining the phenotype of “exhausted” cells and other potential targets underpinning this process, is of obvious practical value [151].

### 6.5. T Regulatory Cells

T regulatory cells (Tregs), commonly defined as cells with phenotype CD45+CD3+CD4+CD25highCD127lowFOXP3, have immunoregulatory functions and suppress the overactivation and proliferation of T effector cells. This is executed via several mechanisms, including the expression of checkpoint ligands (PD1/PD2-L, TIM3-L, LAG3-L, etc.), binding to and blocking of CD80/CD86 activatory ligands on the surface of APC cells (through CTLA-4), trogocytosis of pMHCII from APCs, and competitive capturing of IL-2 via IL2R alpha (CD25) [152]. Based on the origin, Tregs can be divided into two subpopulations, namely thymus-derived Tregs and periphery-derived Tregs. Based on the recent studies, Tregs infiltrating GBM are thymic-derived based on a high expression of chemoattractant ligands such as CCR22 and CCR2 [153,154]. In one study, the presence of Tregs in the peripheral blood in patients with GBM (both recurrent and newly diagnosed) was found to be 2.5-fold higher compared with matching donors [155,156]. At the same time, it seems that their percentage per se in the TME or in the periphery does not correlate with patient survival [157].

The exact mechanisms by which GBM promote Treg infiltration and activity are still to be deciphered. Nevertheless, it was shown recently that perhaps the dysregulation of extracellular glutamate concentration may be the cause [158]. Additionally, in the same study, it was observed that anti-VEGF therapy jeopardizes the immune landscape of the tumor, through the increased transcription from the SLC7A11 gene encoding the glutamate/cystine antiporter and increased extracellular glutamate production promoting Treg activation and CD8+ T cell suppression. Therefore, combinatorial approaches to anti-GBM therapy (preliminary anti-CD25 treatment depleting CD25+ Tregs followed by anti-VEGF treatment) might be more efficient. Notably, Treg cells are capable of surviving in nutrient-scarcity and hypoxic conditions of GBM tumors. It was shown recently [159] that, induced by hypoxia, HIF-1a protein expression in Tregs in the GBM TME is responsible for the active migration of Tregs to the tumor site; however, HIF-1a may inhibit the immunosuppressive function of Tregs by binding to FoxP3 protein. Knockout of HIF-1a led to improved survival of GBM-bearing mice due to a lack of Treg infiltration of the tumor [153,159]. Tregs can be targeted by several (yet, not exclusively Treg-specific) approaches: using anti-PD1, anti-CTLA-4, and anti-CD25 antibodies. However, a low infiltration of CD8+ T cells in the tumor site and low mutation burden in GBM (lack of complementary TCR-MHCI recognition) might limit the application of Tregs as a therapeutic tool. An approach proposed by Amoozgar, Z. et al. [160] directs Treg cells to the GBM TME by engaging glucocorticoid-induced tumor necrosis factor-related protein (GITR) with an agonistic aGITR antibody. Such an approach is based on the fact that GITR expression is significantly higher (~85%) in Tregs form the TME compared with Tregs from the periphery (~25%). The GITR activation in Tregs led to their depletion or reprogramming into effector-like IFN-γ-producing CD4+ T cells. Finally, the high rate of post-treatment relapse of GBM may be explained by the irradiation-induced production of exosomes containing immunosuppressive molecules, such as B7-H4 [161]. That in its turn promotes the expression of FoxP3 in Th1 cells, increasing the immunosuppressive cell population in the TME. Alarmingly, in the case of GBM, Tregs may also promote glioma cells’ stemness [162]. Thereby, Tregs add another layer of complexity to understanding the GBM microenvironment and finding effective treatment strategies.

## 7. B Cells

The essential role of B cells is antigen-specific antibody production. During the tumor progression, the presence of neoantigens promotes priming of B cells and their further differentiation to plasmocytes, promoting antibody-dependent cell cytotoxicity and complement activation, phagocytosis, and antigen neutralization [163]. In addition to conventional B cells, there are so-called B effector cells characterized by the expression of granzymes and perforins, participating in the initiation of direct killing of target cells [164]. All of the above characterize B cells as one of the key participants in anti-tumor immunity. However, several studies postulated that B cells may have controversial activities in tumors, namely promoting Treg differentiation, the production of tumor-nonspecific antibodies, repolarizing macrophages to a more M2-like state, and others [165,166]. Next, recent studies failed to demonstrate that the presence of B cells in the tumor tissue directly correlates with overall survival (OS), depending on the tumor type [163,167].

Many recent studies focus on so-called tertiary lymphoid structures (TLSs). Referring to Laumont et al. [163], TLSs should be defined as the secondary lymphoid structures in non-lymphoid organs, formed by a B cell follicle containing CD21+ follicular dendritic cells (FDCs) and a T cell zone with conventional dendritic cells and high endothelial venules (HEVs). Similarly to natural secondary lymphoid structures, B cells may undergo somatic hypermutation and affinity selection, which can lead to a specific anti-tumor response. According to recent reports, TLS formation is associated with a better prognosis and higher OS for patients with different malignancies [167]. In the case of GBM, there are not many studies about TLSs yet. Nevertheless, Luuk van Hooren et al. [168] analyzed 16 samples from patients with GBM, and CD45+CD3+CD20+ aggregates were found in half of them. The localization of TLSs was mainly in the adjacent meningeal tissue, and tumor-infiltrating T cells’ abundance positively correlated with TLSs’ presence. Interestingly, it was shown that exhausted T cells usually present in GBM express CXCL13, which is a chemokine ligand for B cell recruitment [169]. B cells isolated from the GBM expressed immunosuppressive markers (phenotype PD-L1+CD155+IL10+TGFb+) [166]. Nevertheless, the presence of B cells in different solid tumors is usually associated with a favorable prognosis [166,170,171,172].

## 8. Mesenchymal Cells in GBM TME

### 8.1. Cancer-Associated Fibroblasts

Cancer-associated fibroblasts (CAFs) are fibroblasts that can be found in the TME [173]. They usually play an immunosuppressive role; however, capability of these cells of extracellular matrix (ECM) remodeling and the secretion of anti-inflammatory cytokines (TGFb, IL6) is hijacked by tumor cells. For example, a stiffer ECM prevents immune cell infiltration and may cause vessel collapse, which will lead to hypoxia and limited drug transfer [173,174,175]. They also may participate in the preparation of prometastatic niches and promote the expansion of tumors [173,176]. The major subpopulations of CAFs are myofibroblastic CAFs and non-myofibroblastic CAFs, based on the expression of aSMA (alpha-smooth muscle actin). So-called inflammatory cancer-associated fibroblasts (iCAFs) secrete cytokines and modulate the immune response [173]. The certain origin of CAFs is not yet determined and diverse populations from regional fibroblasts to hematopoietic cells might give rise to CAFs. The major markers of CAFs are as follows: intracellular—aSMA, VIM, and FSP-1; and surface—FAP, PDPN, PDGFRa, and PDGFRb [173]. It was shown that CAFs, via IFN-γ signaling, acquire the ability to express the MHCII complex; however, they lack costimulatory molecules. That leads to an insufficient activation of CD4+ T cells and general immunosuppression [173].

In GBM, CAFs are not fully characterized yet. However, in a recent study of Galbo et al. [177], the CAFs’ presence was revealed in GBM tumor samples close to mesenchymal-like GBM cells in the perinecrotic zone. It has been demonstrated in several studies that CAFs promote chemoresistance in patients with GBM through maintaining the GBM stem-like cell population [178,179]. Additionally, CAFs were found in close proximity to GBM CSCs within a tumor tissue, the cross-talk between CAFs and CSCs was observed, and tumor growth of GSC-derived xenografts was enhanced by CAFs [180]. All of this suggests that CAFs are emerging targets in GBM. The recent review by Galbo Jr timely summarized the roles and possible clinical implications of CAFs in GBM [177].

### 8.2. Mesenchymal Stem Cells

Mesenchymal stem cells (MSCs) are by definition cells of a mesenchymal origin; adhesive during cultivation; capable of differentiation into osteogenic, adipogenic, and chondrogenic lineages; and usually expressing the surface markers CD105, CD73, and CD90 and being CD45- and CD34-negative [181]. Pericytes in the brain seem to possess MSC properties (differentiation capabilities and surface markers) and might be considered as a potential source of MSCs in brain tumors [182,183]. In a recent study of Tumangelova-Yuzeir, Kalina et al. [184], the authors isolated and cultivated GBM-associated MSCs and revealed that besides aforementioned major markers of MSCs, these cells expressed nestin and GFAP—neural markers of differentiation. Therefore, the origin of MSCs in GBM remains unknown. It might be speculated that either MSCs are attracted to the GBM tumor site or these cells are GBM cells directly reprogrammed into GBM-associated MSCs. The role of the CXCR4-CXCL12/SDF1 axis seems to be essential in MSC recruiting along with other chemoattractants such as IL6, VEGF, PDGF, HGF, NT-3, etc., secreted in the GBM TME [185]. The possibility of direct reprogramming of MSCs into GBM CSCs also exists [183]. Finally, it is suggested that MSCs may transdifferentiate into pericytes in the brain tumor site and promote the formation of new vessels [186]. Thus, understanding the origin of MSCs found in GBM may shed light on the origin of GBM. It is also worth mentioning that GBM-derived MSCs promoted Treg proliferation by producing TGFb and IL10 and showed immunosuppressive activity towards Th17 cells [184]. As aforementioned, CD4+ T cells tend to shift to the Th17 phenotype in GBM tumor lesions.

Anti-tumor activities of MSCs are the suppression of angiogenesis through the PDGF-PDGFR axis or FAK axis, suppression of tumor cell proliferation through the inhibition of cyclin D or EGFR expression in tumor cells, and sensitization of the GBM tumor to chemotherapy (described in detail in recent reviews by Nowak, Blazej et al. [185] and Adriana Bajetto et al. [187]). However, MSCs can also promote tumor growth [187,188,189]. Interestingly, the MSC-derived exosome effect on GBM cells depends on the origin of MSCs, and in particular BM-MSC cells suppress tumor proliferation, whereas tumor-associated MSCs (TA-MSCs) promote tumor growth and decrease survival in animal models [190]. Notably, due to MSCs’ high tumor tropism, they are currently used as cellular vehicles of different anti-tumor drugs [191], and the same approach can be used in the case of GBM. However, MSCs should be “improved” to be retained in the tumor tissue for a prolonged period of time. For that purpose, the formation of MSC spheroids can be used [192].

## 9. Conclusions

GBM is characterized by a staggering heterogeneity of the cell populations within its TME, both inter- and intra-patient. It is suggested that the overall composition of such populations comprising the tumor mass, the percentage of particular types of cells (both resident and transient), their spatiotemporal dynamic within the TME, and the activity and phenotype might vary significantly based on GBM grade, localization, type and stage of therapy, and so on and so forth. As discussed in the current review, the cancer cells in GBM, including CSCs, are dependent on and shaped by the interplay with non-tumor cells within the TME (the concept of so-called tumor “ecosystem”) [193]. Thus, a detailed characterization of non-tumor cell populations in the GBM TME is needed to choose a compelling curative approach, as all of them contribute to the pathogenesis and treatment response. Finally, the “response” of all populations within the TME should be assessed during and after the treatment, to decipher molecular mechanisms underlying resistance to therapy and disease relapse.

## Data Availability

Not applicable.
